# Fragility of cardiovascular outcome trials (CVOTs) examining nutrition interventions among patients with diabetes mellitus: a systematic review of randomized controlled trials

**DOI:** 10.1007/s42000-022-00396-5

**Published:** 2022-09-21

**Authors:** Niki Taouktsi, Stefanos T. Papageorgiou, Georgios Tousinas, Stavroula Papanikolopoulou, Maria G. Grammatikopoulou, George Giannakoulas, Dimitrios G. Goulis

**Affiliations:** 1 Medical School, Faculty of Health Sciences, Aristotle University of Thessaloniki, Thessaloniki, Greece; 2 Unit of Reproductive Endocrinology, 1st Department of Obstetrics and Gynecology, Medical School, Aristotle University of Thessaloniki, Papageorgiou General Hospital, Thessaloniki, GR-56429 Greece; 3 Department of Rheumatology and Clinical Immunology, Medical School, University of Thessaly, Larissa, Greece; 4 Department of Cardiology, AHEPA University Hospital, Aristotle University of Thessaloniki, Thessaloniki, Greece

**Keywords:** Statistical robustness, RCT, Dietary supplements, Cardiovascular disease, Research methodology, Meta-epidemiology, Fragility quotient

## Abstract

**Purpose:**

There is controversy regarding the optimal statistical method to interpret how robust is a statistically significant result. The fragility index (FI) and the reverse fragility index (RFI) are quantitative measures that can facilitate the appraisal of a clinical trial’s robustness. This study was performed to evaluate the FI and RFI of randomized controlled trials (RCTs) examining nutritional interventions in patients with diabetes mellitus, focusing on cardiovascular outcomes.

**Methods:**

A systematic search was conducted and relevant RCTs were identified in three databases. RCTs examining nutritional interventions (supplements or dietary patterns) in patients with DM with dichotomous primary endpoints involving cardiovascular outcomes were eligible. Data were extracted to compose 2 × 2 event tables and the FI and RFI were calculated for each comparison, using Fisher’s exact test. Risk of bias (RoB) of the included RCTs was assessed with the Cochrane RoB 2.0 tool.

**Results:**

A total of 14,315 records were screened and 10 RCTs were included in the analyses. The median FI of the paired comparisons was 3 (IQR: 2–4) and the median RFI was 8 (IQR: 4.5–17). RoB and heterogeneity were low.

**Conclusions:**

RCTs examining nutritional interventions and cardiovascular outcomes among patients with diabetes mellitus appear to be statistically fragile. Τhe FI and the RFI can be reported and interpreted as an additional perspective of a trial’s robustness.

**Highlights:**

• In the evidence-healthcare era, assessing how robust statistically significant results are remains a matter of controversy.

• Recently, the fragility index (FI) and reverse fragility index (RFI) were proposed to assess the robustness of randomized controlled trials (RCTs) with 2 × 2 comparisons.

• When applying the FI and RFI, RCTs examining nutritional interventions and cardiovascular outcomes among patients with diabetes mellitus (DM) appear to be statistically fragile.

• Τhe FI and the RFI can be reported and interpreted as an additional perspective of a trial’s robustness.

• RCTs implementing nutrition interventions among patients with DM can improve their methodology.

## Introduction

Globally, it is estimated that 463 million adults are living with diabetes mellitus (DM), with the projected number being expected to reach 700 million by the year 2045 [1, 2]. Approximately 10% of all people with DM have type 1 DM, while type 2 diabetes (T2DM) constitutes the most common form, accounting for the majority (90%) of all cases worldwide [3]. Medical nutrition therapy (MNT) composes a fundamental, cost-effective component of quality DM care, decelerating complications and improving quality of life [4–6]. Heart disease-specific mortality is 2–4 times higher among adults with DM compared with DM-free adults [7], and all types of DM have been shown to multiply the risk of atherosclerotic vascular disease and the burden of cardiovascular disease (CVD), in general [1, 8–11]. Consequently, research has long focused on possible dietary interventions for the joint prevention of DM and CVD, using cardiovascular outcome trials (CVOTs).

Today, living as we are during the era of evidence-based medicine, clinical decision-making is based on research evidence of ever higher hierarchy, with primary evidence stemming mainly from randomized control trials (RCTs), as they are considered the gold standard in establishing guideline recommendations [12]. Traditionally, the results of clinical trials are evaluated with measures such as statistical significance (*p*-value) and confidence intervals [13]. However, more recently, a controversy arose concerning the sufficiency of such statistics to interpret the robustness of a study’s outcome [14, 15].

A quantitative measure, the fragility index (FI), was proposed to aid researchers in appraising the robustness of statistical significance in 2 × 2 event tables [16]. The FI is defined as the minimum number of participants whose outcome would have to change from an event to a non-event, i.e., to cause a statistically significant result to become non-significant. The lower the FI, the less robust is the result [16, 17].

However, the FI is exclusively applied to trials that reach traditional statistical significance. In the opposite case of statistically non-significant results, the reverse fragility index (RFI) can be calculated instead. In contrast to the FI, the RFI represents the minimum number of events needed to reverse a non-significant result to a significant one [18]. By definition, the FI is only appropriate for dichotomous outcomes and cannot be applied to continuous variables due to its method of calculation [16]. To further understand the notion of the FI relative to sample size, the fragility quotient (FQ) can be calculated by dividing FI by sample size [16]. Accordingly, the reverse fragility quotient (RFQ) is calculated by dividing the RFI by each trial’s total N.

Although nutrition is a known and established effector of both DM and CVD, nutrition trials have frequently been questioned regarding their methodology and robustness [19]. Meta-epidemiological studies have pointed to the overall mediocrity of the FI among clinical nutrition trials [20] (median FI: 1, range: 1–3), while concerning interventions promoting the Mediterranean diet (MD), similar findings were observed (median FI: 5, range: 1–39; median RFI: 7, range: 1–29) [21].

The purpose of the present meta-epidemiological study was to systematically review all RCTs assessing the robustness of CVOTs examining nutritional interventions among patients with DM and estimate their FI or RFI.

## Methods

### Research question, PICO, and protocol registry

The search question of the study was the following: what is the FI and RFI of RCTs assessing the effects of nutritional interventions on cardiovascular outcomes among patients with diabetes mellitus? The PICO format of the research question is presented in Table 1.

**Table 1:** PICO format of the study’s research question

PICO components	Determinants
(P)opulation:	Adults with diabetes mellitus type 1 or 2
(I)ntervention:	Any type of nutritional intervention (micro/macronutrients, dietary supplements, integrated nutritional standards)
(C)omparator:	Any other type of nutritional intervention or usual diet
(O)utcome:	Vascular events (atherosclerosis, total cardiovascular risk, stroke, coronary artery disease, peripheral artery disease, sudden death)

The present systematic review was registered at the Center for Open Sciences (OSF) (https://bit.ly/3aE2zTu) and followed the Preferred Reporting Items for Systematic Reviews and Meta-Analyses (PRISMA) guidelines [22].

### Search strategy

A systematic literature search was conducted by four researchers (N.T., S.T.P., G.T., and S.P.) independently in the following databases: PubMed, Scopus, and the Cochrane rEgister of controlled TRiALs (CENTRAL), from inception until July 2020. Furthermore, studies were also identified from the gray literature. Rayyan [23], a web and mobile app for systematic reviews, was used by four independent researchers (N.T., S.T.P., G.T. and S.P.) for the scanning and identification of RCTs fulfilling the study’s criteria. Cited references identified were imported to Rayyan and duplicate entries were removed. A more experienced researcher (D.G.G.) provided advice whenever required.

**Fig. 1:**
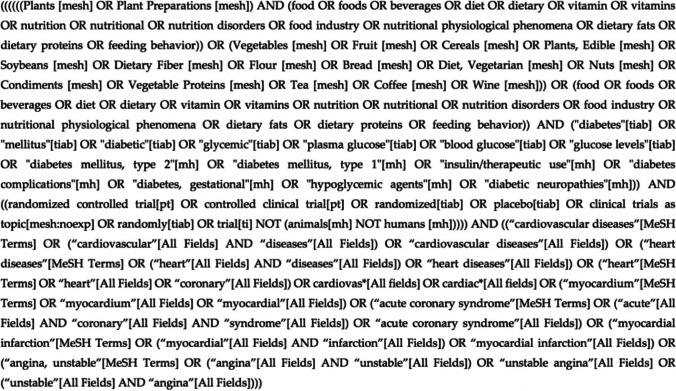
Search string used for the PubMed database

The search terms used included a combination of the population (DM), the intervention (diet), and the outcome (CVD) as recommended by the Cochrane Collaboration Handbook. An example of the search string applied on PubMed is presented in Fig. 1.

### Inclusion and exclusion criteria

The inclusion criteria for the study involved the following: studies (1) with an RCT design, (2) with dichotomous primary outcomes, (3) including adult patients with DM, (4) with dietary intervention (dietary supplements, dietary pattern), (5) compared against a non-dietary intervention, sham diet, no intervention, or placebo, (6) assessing any primary cardiovascular outcome, and (7) with results based on a 2 × 2 events table, and 8) research items without any restrictions on the published language.

Exclusion criteria included the following: studies (1) lacking an RCT design, (2) with continuous primary outcomes, (3) lacking participants with DM, (4) without a 2 × 2 events table, or data in order to produce a 2 × 2 events table, and (5) performed on animals or children.

### Risk of bias

The Cochrane Risk of Bias (RoB) Tool 2.0 [24] was applied by two reviewers (N.T. and S.T.P.) independently to evaluate selected RCTs for potential sources of bias. When different opinions arose, another researcher aided the decision (D.G.G.)

### Data extraction

Two reviewers working independently (N.T. and M.G.G.) extracted data, and disagreements were arbitrated by a senior team member (D.G.G.). Data were extracted using a pilot electronic form for the following variables: first author, country of conduct, year of publication, RCT design, sample size, type of intervention(s) and comparison(s), duration of the intervention, primary outcome(s), number of participants and events at each arm, *P*-value of each comparison, and masking.

### Calculation of the fragility index, reverse fragility index, fragility quotient, and reverse fragility quotient

The FI of each outcome was calculated based on the method originally described by Wash et al. [16]. The results of each eligible study were placed in a 2 × 2 contingency table. One positive result (event) was added to the group with the smaller number of positive outcomes, while one negative result (non-event) was subtracted from the same group to keep the total number of patients constant.

Statistical significance (*P*-value) was recalculated using Fisher’s exact test. This procedure was repeated until the calculated *P*-value exceeded 0.05. The number of additional positive outcomes required to reach a *P*-value of greater than 0.05 was considered the trial’s FI.

In the case of non-significant results, the RFI was calculated instead by subtracting events from the group with the smaller number of events while simultaneously adding non-events to the same group in order to maintain the total number of participants constant until the Fisher’s exact test *P*-value became less than 0.05 [18, 21].

Moreover, the FQ [25] was calculated for each trial with a significant comparison by dividing the FI score by the total study sample size. On the other hand, the RFQ was calculated for all non-significant comparison arms.

Given that the FI and RFI are only applicable on 2 × 2 tables, each intervention was compared against the comparator/placebo arm separately, and the FI/RFI was calculated in trials with more than one intervention.

Microsoft Excel® was used for the calculation of the FIs and RFIs for each comparison of the selected trials. Both measures were calculated for the DM subgroups and the total number of participants of each trial.

### Statistical analyses

Group differences in the FIs and RFIs were assessed with the Mann–Whitney U test. The Jamovi project (Version 0.9.5.16) was used for these analyses. The level of significance was set at 0.05 unless otherwise specified.

Heterogeneity of the comparisons included in the analyses was assessed with the Q statistic [26] and the use of a random effects model in Review Manager [27].

## Results

### Selection of trials

**Fig. 2:**
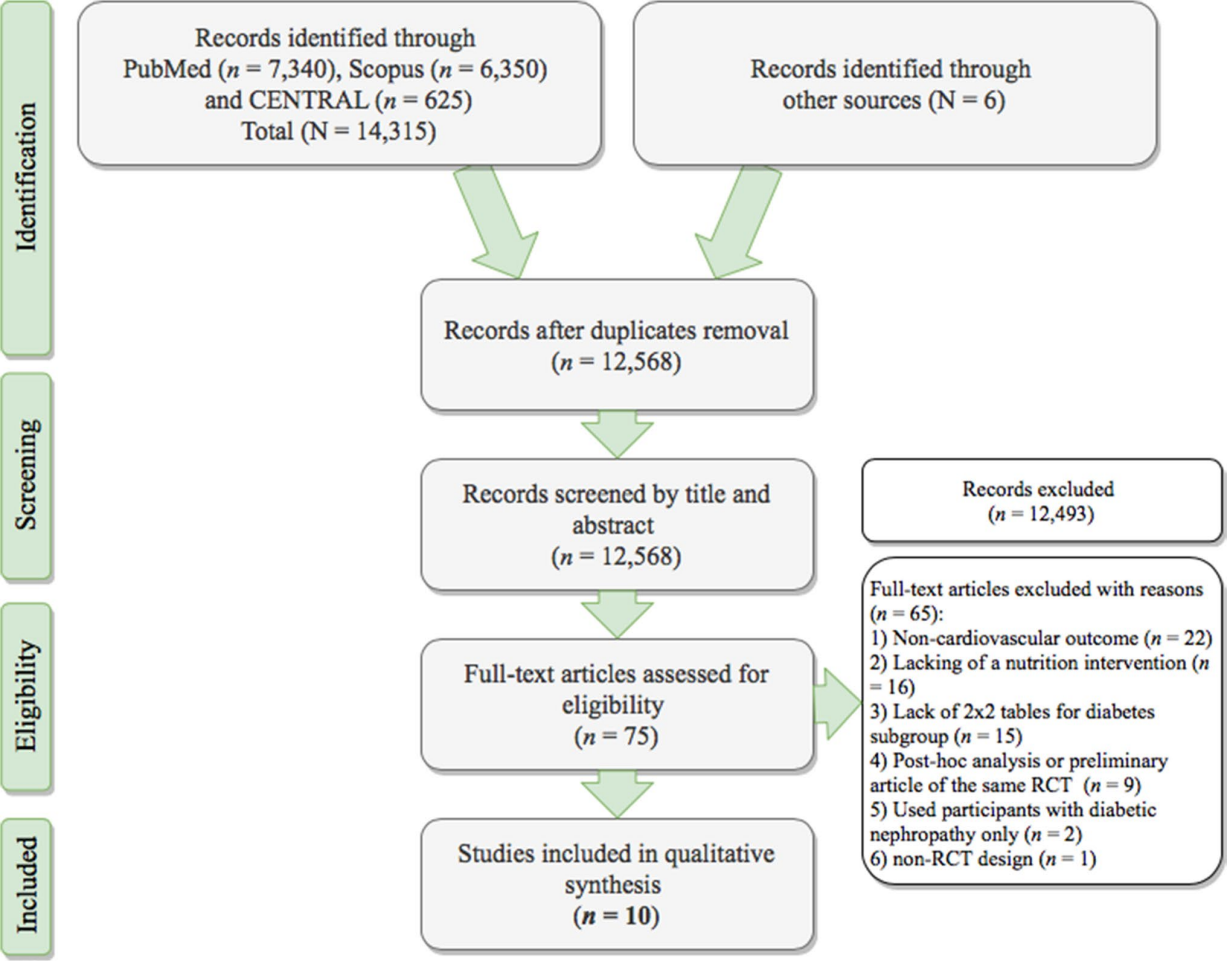
PRISMA flowchart of the studies selection process

The initial search identified 14,315 records, of which 78 met the prespecified inclusion criteria to be assessed through a full-text review. The primary reason for exclusion was the lack of a dichotomous outcome. A total of 10 RCTs were included in the final quantitative analysis. Figure 2 presents the flow diagram outlining the selection of studies according to Preferred Reporting Items for Systematic Reviews and Meta-Analyses (PRISMA) guidelines.

### Trial characteristics, interventions, and outcomes

The characteristics of the included RCTs are presented in Table 2. All trials were of parallel design. Two trials originated from the UK [28, 29], one each was conducted in Spain [30], the USA [31], Italy [32], the Netherlands [33], Finland [34], and Israel [35], and two were multi-country trials [36, 37].

**Table 2:** Characteristics of the RCTs included in the analyses (DM subgroups)

Trial acronym	First author	Multicenter	Masking	Design	Origin	Randomization	Population	Intervention(s) and comparator(s)	Intervention duration	Primary outcome(s)	Registry
Alpha-Omega	Kromhout [33]	√	Double-blind	2 × 2 factorial	Netherlands	NR	*Ν* = 1014 patients with DM (60–80 yrs) with a history of MI < 10 yrs prior to randomization	1. EPA + DHA (400 mg/d) (*n* = 262) 2. ALA (2 g/d) (*n* = 258) 3. EPA + DHA (400 mg) + ALA (2 g) (*n* = 245) 4. Placebo (*n* = 249)	40 mo	Ventricular arrhythmia-related events and fatal MI	NCT00127452
ASCEND	ASCEND Collaborative Group [28]	√	Double-blind	Prospective parallel	UK	minimized	N = 15,480 patients with DM (≥ 40 yrs), CVD-free	1. marine n − 3 FA (460 mg EPA + 380 mg DHA) (*n* = 7740) 2. placebo caps (olive oil) (*n* = 7740)	7.4 yrs (median)	First serious vascular event (a composite of non-fatal MI or stroke, TIA, or vascular death)	NCT00135226
ATBC	Kataja-Tuomola [34]	-	Double-blind	2 × 2 factorial	Finland	By blocks of 8 within each of the 14 study sites	*N* = 1700 male smokers with DM (subgroup) (50–69 yrs)	1. α-tocopherol (50 mg/d) (*n* = 417) 2. β-carotene (20 mg/d) (*n* = 434) 3. α–tocopherol (50 mg) + β-carotene (20 mg) (*n* = 443) 4. Placebo (*n* = 406)	6.1 yrs (median)	Macrovascular outcomes (major coronary event, stroke, PAD) and deaths	NR
Heart protection Study	Heart Protection Study Collaborative Group [29]	√	Double-blind	2 × 2 factorial	UK	central phone randomization with a minimization algorithm	*N* = 5963 patients with DM (subgroup) (40–80 yrs) with history of CHD, OAD, DM, hypertension	1. ONS with vitamin E (600 mg) + vitamin C (250 mg) + β-carotene (20 mg) (*n* = 2981) 2. Placebo (*n* = 2,982)	5 yrs (median)	5-yr mortality, or incidence of any vascular disease, cancer, or other major outcomes	NR
I CARE	Milman [35]	√	Double-blind	Prospective parallel	Israel	PC-generated	*N* = 1434 patients with T2DM (≥ 55 yrs) and the Hp 2–2 genotype	1. Vitamin E (400 IU/d) (*n* = 726) 2. Placebo (*n* = 708)	18 mo	Composite of CV death, non-fatal MI, and stroke	NCT00220831
PREDIMED	PREDIMED Study Investigators [30]	√	Single-blind	Prospective parallel	Spain	PC-generated random-number sequence	N = 3614 patients with T2DM (no CVD at enrollment) or ≥ 3 risk factors (smoking, high LDL, hypertension, low HDL, overweight/obesity, family history of premature CHD) (men 55–80 yrs, women 60–80 yrs)	1. MD with EVOO (*n* = 1282) 2. MD with nuts (*n* = 1143) 3. Control diet (*n* = 1189)	4.8 yrs (median)	Composite of MI, stroke, and death from CV causes	SRCTN35739639
REDUCE-IT	Bhatt [36]	√	Double-blind	Prospective parallel^†^	MC	by CV risk stratum, ezetimibe use, geographic region	*N* = 4787 patients with DM (subgroup), ≥ 50 yrs and an additional risk factor for CVD	1. E-EPA^‡^ (2 × 2 g/d with food) (*n* = 2394) 2. Placebo (*n* = 2393)	4.9 yrs (median)	Composite of CVD death, non-fatal MI (or silent MI), non-fatal stroke, unstable angina, coronary revascularization	NCT01492361
Risk & Prevention (R&P)	The Risk and Prevention Study Collaborative Group [32]	√	Double-blind	Prospective parallel	Italy	by phone, based on a concealed, PC-generated list, stratified by GP	N = 7494 patients with DM (subgroup) + 1 risk factor (obesity, > 65 yrs, hypertension, hypercholesterolemia, male sex, premature CVD family history, smoking)	1. n–3 fatty acids* (*n* = 3721) 2. placebo (*n* = 3773)	5 yrs (median)	Cumulative of death, non-fatal MI, non-fatal stroke, revised at 1 yr as the composite of time-to-death or hospitalization for CV	NCT00317707
TIDE	The TIDE Trial Investigators [37]	√	Double blind	3 × 2 factorial	MC (33 countries)	central phone-in PC-system	*N* = 1221 patients with T2DM, HbA1c: 6.5–9.5%, at risk of CVD	1. Vitamin D (1,000 IU) (*n* = 607) 2. Placebo (*n* = 614)	130 d	All-cause death or cancers requiring surgery, hospitalization, or chemotherapy	NCT00879970
WAFACS	Albert [31]	-	Double-blind	4-arm factorial	USA	5-yr age groups	*N* = 1144 women with DM (subgroup), ≥ 40 yrs, post-menopausal or not intending to be pregnant, CVD history, or > 3 cardiac risk factors	1. ONS with folic acid (2.5 mg), vitamin B_6_ (50 mg), vitamin B_12_ (1 mg) (*n* = 570) 2. Placebo (*n* = 574)	7.3 years (median)	Combined endpoint of CVD and mortality (MI, stroke, coronary revascularization procedures)	NCT00000541

*ASCEND*, A Study of Cardiovascular Events in Diabetes; *ASA*, acetylsalicylic acid; *ATBC,* Alpha-Tocopherol, Beta-Carotene Cancer Prevention Study; *BW,* body weight; *CAD*, coronary artery disease; *CHD*, Coronary heart disease; *CV,* cardiovascular; *CVD*, cardiovascular disease; *DHA*, docosahexaenoic acid; *DM*, diabetes mellitus; *E-EPA*, ethyl eicosapentaenoic acid; *EDTA-Na*_*2*_, edetate disodium; *EPA*, eicosapentaenoic acid; *EVOO*, extra-virgin olive oil; *FA*, fatty-acids; *GP*, general practitioner; *HbA1c*, glycosylated haemoglobin; *HDL*, high-density lipoprotein; *Hp*, Haptoglobin; *IV*, intravenous; *IU*, international units; *MC*, multi-country; *MD*, Mediterranean diet; *MI*, myocardial infarction; *MgCl*_*2*_, magnesium chloride; *NR*, not reported; *PA*, physical activity; *PAD*, peripheral artery disease; *PC*, personal computer; *OAD*, occlusive arterial disease; *ONS*, oral nutrient supplementation; *PREDIMED*, Prevención con Dieta Mediterránea; *PRO*, protein; *REDUCE-IT*, reduction of cardiovascular events with icosapent ethyl–intervention trial; *T2DM*, type 2 diabetes mellitus; *TC*, total cholesterol; *TIA*, transient ischemic attack; *TIDE*, thiazolidinedione intervention with vitamin D evaluation; *WAFACS,* the Women’s Antioxidant and Folic Acid Cardiovascular Study; ^†^ phase 3b trial; ^‡^ highly purified and stable EPA ethyl ester; ^^^ the n of participants in this group was not reported for the DM subgroup; * poly-unsaturated fatty acid ethyl esters with EPA and DHA content not < 85%, in a ratio that could range from 0.9:1 to 1.5:1

The median sample size restricted to patients with DM was 2657. The total sample sizes of the included trials ranged between 1014 [33] and 15,480 [38]. The median total number of events across both treatment groups for all outcomes was 23 (IQR, 7.75–84). Most RCTs had double-blind masking [28, 29, 31–37] and one was single-blind [30].

The performed interventions involved dietary supplements with fatty acids [28, 32, 33, 36] or vitamins [29, 31, 34, 35, 37], or adherence to the MD pattern with either extra-virgin olive oil (EVOO) or nuts [30]. More than one intervention was offered in three trials [28, 30, 34], with the remaining having one intervention and one comparator arm only.

Most trials examined composite primary outcomes of major vascular events (myocardial infarction, stroke, or CV death) [28, 30, 31, 34–36]. Other outcomes included individual CV outcomes, including coronary events [28, 34], CVD [29], stroke [34], myocardial infarction [33], coronary revascularization, transient ischemic attack (TIA) [28], peripheral artery disease (PAD) [34], ventricular arrhythmia-related events [33], hospital admission for CV causes [37], CV [29, 34], and all-cause [37] mortality. A few RCTs also had cancer occurrence as an endpoint, which is also considered an integral component of a CVOT design [39, 40]. The majority of them were based on a time-to-event analysis, with only one outcome being adjusted.

The reported *P*-values for each outcome exceeded 0.05 82% of the comparison, less than 0.05 for 6 (12%), less than 0.01 for 2 (4%) and less than 0.001 for 1 (2%).

### Heterogeneity of the comparisons

For the DM subgroups, a total of 24,123 patients with DM were pooled for the calculation of the FI and the FQ, and 137,815 patients were pooled for the calculation of the RFI and the RFQ. Heterogeneity was calculated at 63 and 0% for the FI and RFI, respectively.

For the calculation of the FI and the FQ in the total number of participants of each study, a total sample of 27,021 participants was used (13,526 participants in the intervention arms and 13,495 controls), and the calculated heterogeneity was 70%, based on six comparisons. For the RFI and RFQ calculations based on the total number of participants in each RCT, 467,193 participants were used in total and the calculated I^2^ was 0%.

### Risk of bias

**Fig. 3:**
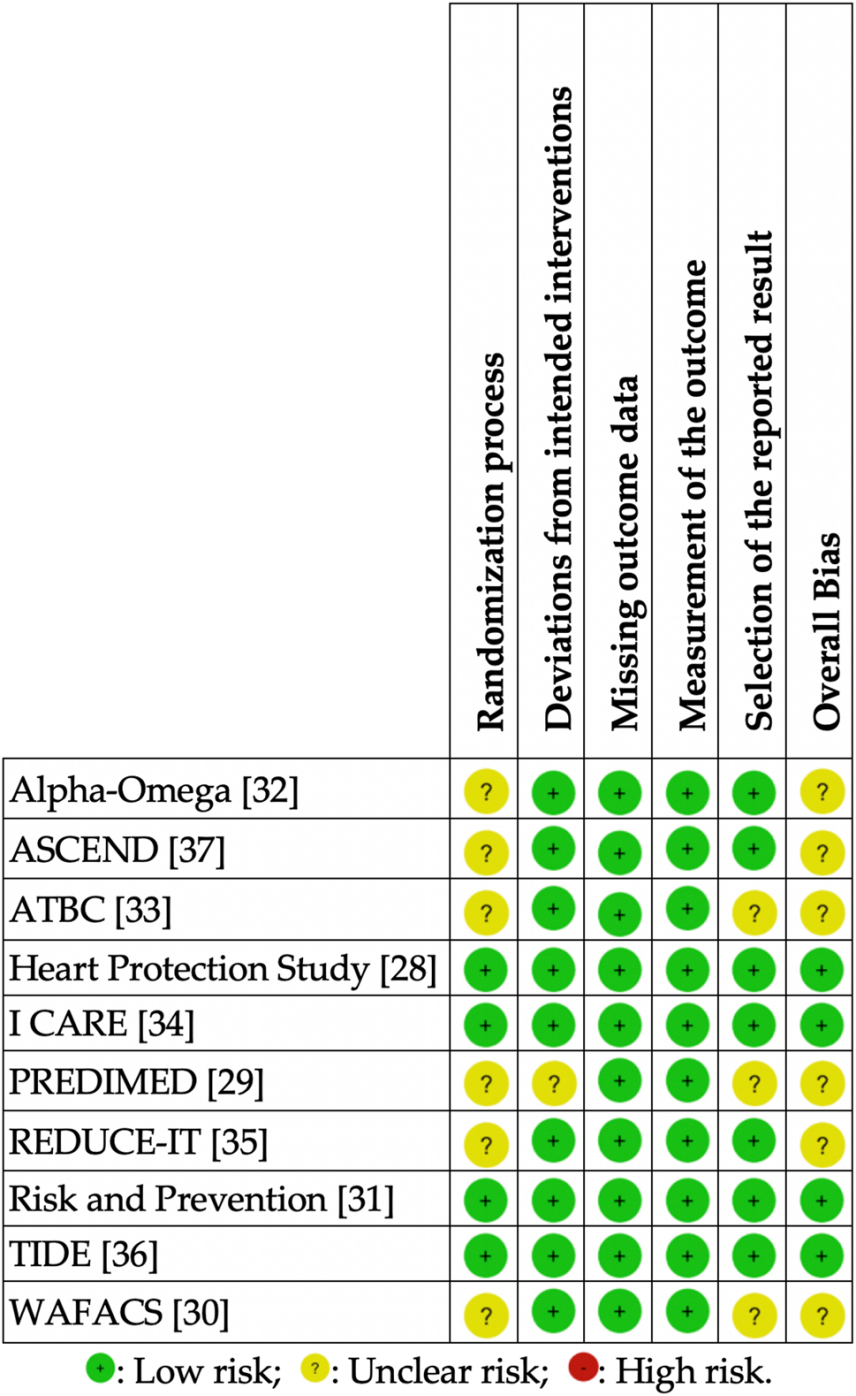
Risk of Bias of the included RCTs. *ASCEND*, A Study of Cardiovascular Events in Diabetes; *ATBC*, Alpha-Tocopherol, Beta-Carotene Cancer Prevention Study; *PREDIMED*, Prevención con Dieta Mediterránea; *RCT*, randomized controlled trial; *REDUCE-IT*, Reduction of Cardiovascular Events with Icosapent Ethyl–Intervention Trial; *RoB*, Risk of bias [24]; *TIDE*, thiazolidinedione intervention with vitamin D evaluation; *WAFACS*, the Women’s Antioxidant and Folic Acid Cardiovascular Study

Cochrane risk of bias assessment is summarized in Fig. 3. The majority of trials (60%) aroused some concerns regarding the overall risk of bias, mainly due to the randomization process. Most of them were deemed of low risk of bias concerning the deviations from the intended intervention (90%), missing outcome data (100%), outcome measurement (100%), and selective reporting (70%). None of them was considered an overall high risk of bias.

### FIs, RFIs, FQs, and RFQs of the RCTs (DM subgroups)

Table 3 reports the FIs and RFIs of the included RCTs for the subgroups of patients with DM. In four (40%) [28, 33, 35, 36] out of 10 trials in total, at least one statistically significant outcome was calculated, all with the significance level set at *P* ≤ 0.05. Of the 49 paired comparisons, six (12%) were statistically significant and their FI was calculated.

**Table 3:** Calculation of the Fragility Index and Reverse Fragility of the included RCTs (DM subgroup)

Trial acronym	Comparison	Primary outcome(s)	Intervention arm (*n*)	Comparator arm(*n*)	Events in intervention (*n*)	Events in comparator (*n*)	Significance (*P*-value)	FI	RFI	FQ	RFQ
Alpha- Omega [33]	EPA–DHA *vs*. placebo	Ventricular arrhythmia events	262	249	8	13	0.267	-	4		0.008
		Death from MI	262	249	5	7	0.568	-	4		0.008
		Ventricular arrhythmia events, or MI death	262	249	13	20	0.207	-	4		0.008
		All-cause mortality	262	249	26	31	0.400	-	8		0.016
	ALA *vs*. placebo	Ventricular arrhythmia events	258	249	6	13	0.103	-	2		0.004
		Death from MI	258	249	11	7	0.474	-	5		0.010
		Ventricular arrhythmia events or MI death	258	249	17	20	0.610	-	8		0.016
		All-cause mortality	258	249	28	31	0.583	-	11		0.022
	EPA–DHA + ALA *vs*. placebo	Ventricular arrhythmia events	245	249	2	13	0.007	3	-	0.006	
		Death from MI	245	249	4	7	0.544	-	4		0.008
		Ventricular arrhythmia events, or MI death	245	249	6	20	0.008	3	-	0.006	
		All-cause mortality	245	249	25	31	0.479	-	8		0.016
ASCEND [28]	n − 3 fatty acids *vs*. placebo	Non-fatal MI	7740	7740	186	200	0.503	-	24		0.001
		Non-fatal ischemic stroke	7740	7740	217	214	0.922	-	37		0.002
		TIA	7740	7740	185	180	0.832	-	37		0.002
		Vascular death	7740	7740	186	228	0.041	2	-	0.0001	
		Serious vascular event	7740	7740	689	712	0.538	-	47		0.003
		Any revascularization	7740	7740	368	356	0.675	-	40		0.003
		Serious vascular event/revascularization	7740	7740	882	887	0.919	-	73		0.005
ATBC [34]	α-tocopherol ONS *vs*. placebo	Total outcomes	417	406	51	52	0.834	-	16		0.019
		Major coronary events	417	406	21	30	0.193	-	5		0.006
		Total stroke	417	406	16	13	0.707	-	8		0.010
		PAD	417	406	14	9	0.399	-	5		0.006
		Total mortality	417	406	78	72	0.787	-	17		0.020
	β-carotene ONS *vs*. placebo	Total outcomes	434	406	74	52	0.100	-	4		0.004
		Major coronary events	434	406	41	30	0.321	-	8		0.010
		Total stroke	434	406	25	13	0.096	-	2		0.002
		PAD	434	406	8	9	0.808	-	6		0.007
		Total mortality	434	406	84	72	0.594	-	15		0.018
	a-tocopherol + β-carotene ONS *vs*. placebo	Total outcomes	443	406	54	52	0.836	-	20		0.024
		Major coronary events	443	406	28	30	0.587	-	10		0.012
		Total stroke	443	406	18	13	0.584	-	7		0.008
		PAD	443	406	8	9	0.807	-	6		0.007
		Total mortality	443	406	84	72	0.658	-	17		0.020
Heart Protection Study [29]	ONS with vitamins E, C and β-carotene *vs*. placebo	First major vascular event	2981	2982	663	686	0.496	-	41		0.007
I CARE [35]	Vitamin E *vs*. placebo	Primary composite	726	708	16	33	0.013	4	-	0.003	
		MI	726	708	7	17	0.039	1	-	0.001	
		Stroke	726	708	6	11	0.230	-	3		0.002
		CV death	726	708	3	5	0.501	-	3		0.002
PREDIMED[30]	Combined diets *vs*. usual diet	Rate of major CV events	2425	1189	121	69	0.303	-	17		0.005
REDUCE-IT [36]	E-EPA *vs.* placebo	Composite of CV death, non-fatal MI/stroke, unstable angina, coronary revascularization	2394	2393	433	536	0.001	48	-	0.010	
R&P [32]	n-3 fatty acids *vs*. placebo	Risk of death or 1^st^ hospitalization for CV	3720	3772	439	458	0.669	-	42		0.006
TIDE [37]	Vitamin D *vs*. placebo	Primary CV outcome	607	614	2	3	1.000	-	7		0.006
		CV death	607	614	0	1	1.000	-	2		0.002
		Non-fatal MI	607	614	1	1	1.000	-	6		0.005
		Non-fatal stroke	607	614	1	1	1.000	-	6		0.005
		Any revascularization	607	614	5	7	0.773	-	5		0.004
		Hospitalization for heart failure	607	614	2	0	0.247	-	3		0.002
WAFACS [31]	ONS with folic acid, vitamins B_12_ and B_6_ *vs*. placebo	Combined endpoint of CVD and mortality	570	574	142	143	1.000	-	28		0.024

*ALA,* a-linolenic acid; *ASCEND*, A Study of Cardiovascular Events in Diabetes; *ATBC,* Alpha-Tocopherol, Beta-Carotene Cancer Prevention Study*; CV,* cardiovascular; *CVD*, cardiovascular disease; *DHA*, docosahexaenoic acid; *EPA*, eicosapentaenoic acid; *E-EPA*, Ethyl eicosapentaenoic acid; *FI*, fragility index; *FQ*, Fragility quotient; *MI*, Myocardial infarction; *ONS*, oral nutrient supplementation; *PAD*, Peripheral artery disease; *PREDIMED*, Prevención con Dieta Mediterránea; *REDUCE-IT*, Reduction of Cardiovascular Events with Icosapent Ethyl–Intervention Trial*; RCT*, randomized controlled trial; *RFI*, reverse fragility index; *RFQ*, reverse fragility quotient; *R&P,* risk and prevention; *TIA*, transient ischemic attack; *TIDE*, the Thiazolidinedione Intervention with vitamin D Evaluation; *WAFACS,* the Women’s Antioxidant and Folic Acid Cardiovascular Study

Two trials failed to report the number of patients with DM lost to follow-up [33, 34]. The median FI for the six statistically significant comparisons was 3 (range: 1–48, IQR 2–4). In four (67%) out of six comparisons with a significant outcome, the FI was ≤ 3. The median FQ of the included trials was 0.004 (range: 0.001–0.100, IQR: 0.001–0.006).

The RFI was calculated for all non-statistically significant comparisons (43 in total). The median RFI was 8 (range: 2–73, IQR 4.5–17). In 6 (14%) out of 43 different comparisons, the calculated RFI had a value of ≤ 3. The median RFQ was 0.007 (range: 0.002–0.024, IQR 0.004–0.011).

In 75% of the trials, the number of patients lost to follow-up was greater than the calculated FI.

### FIs, RFIs, FQs, and RFQs based on the total sample of participants of each RCT

In 50% of the included RCTs, the total study population was different from the DM subgroup. One of the trials reported CV outcomes only for patients with DM [34]. Considering that allocation to the intervention was performed regardless of DM status, the FIs and RFIs were also calculated for the total sample randomized in each trial to reduce the risk of bias arising from interfering with the randomization procedure.

The median sample size of the total study populations used in the RCTs was 7447, ranging between 1014 and 20,536, with a median of 205 events (range: 1–4618) and a median follow-up duration of 4.95 years.

Table 4 reports the FIs and RFIs of each trial calculated for the primary outcomes of the total study population. In four (40%) out of 10 trials in total [28, 30, 33, 35], at least one statistically significant comparison was apparent, involving six paired comparisons in total and the FIs were calculated accordingly. Two of the trials [33, 34] did not report the number of patients lost to follow-up. The median number of participants lost to follow-up was 74.5 (range: 2–523, IQR: 32.75–214.25).

**Table 4:** Calculation of the Fragility Index and Reverse Fragility of the included RCTs (total study population)

Trial acronym	Comparison	Primary outcome(s)	Intervention arm (***n***)	Comparatorarm (***n***)	Events in intervention(***n***)	Events in comparator(***n***)	Significance (***P***-value)	FI	RFI	FQ	RFQ
Alpha- Omega [33]	EPA–DHA *vs*. placebo	Ventricular arrhythmia events	262	249	8	13	0.267	-	4		0.008
		Death from MI	262	249	5	7	0.568	-	4		0.008
		Ventricular arrhythmia events, or MI death	262	249	13	20	0.207	-	4		0.008
		All-cause mortality	262	249	26	31	0.400	-	8		0.016
	ALA *vs*. placebo	Ventricular arrhythmia events	258	249	6	13	0.103	-	2		0.004
		Death from MI	258	249	11	7	0.474	-	5		0.010
		Ventricular arrhythmia events or MI death	258	249	17	20	0.610	-	8		0.016
		All-cause mortality	258	249	28	31	0.583	-	11		0.022
	EPA–DHA + ALA *vs*. placebo	Ventricular arrhythmia events	245	249	2	13	0.007	3	-	0.006	
		Death from MI	245	249	4	7	0.544	-	4		0.008
		Ventricular arrhythmia events, or MI death	245	249	6	20	0.008	3	-	0.006	
		All-cause mortality	245	249	25	31	0.479	-	8		0.016
ASCEND [28]	n − 3 fatty acids *vs*. placebo	Non-fatal MI	7740	7740	186	200	0.503	-	24		0.002
		Non-fatal ischemic stroke	7740	7740	217	214	0.922	-	37		0.002
		TIA	7740	7740	185	180	0.832	-	37		0.002
		Vascular death	7740	7740	186	228	0.041	2	-	0.0001	
		Serious vascular event	7740	7740	689	712	0.538	-	47		0.003
		Any revascularization	7740	7740	368	356	0.675	-	40		0.003
		Serious vascular event/revascularization	7740	7740	882	887	0.919	-	73		0.005
Heart Protection [29]	ONS with vitamins E, C and β-carotene *vs*. placebo	Coronary cause of death	10,269	10,267	664	630	0.343	-	35		0.002
		Other vascular cause of death	10,269	10,267	214	210	0.883	-	36		0.002
		Any vascular cause of death	10,269	10,267	878	840	0.351	-	40		0.002
		Major coronary event	10,269	10,267	1,063	1,047	0.730	-	70		0.003
		Non-fatal MI	10,269	10,267	464	467	0.920	-	54		0.003
		Non-fatal stroke	10,269	10,267	430	435	0.862	-	50		0.002
		Fatal stroke	10,269	10,267	108	107	1.000	-	27		0.001
		Any stroke	10,269	10,267	511	518	0.823	-	53		0.003
		Coronary revascularization	10,269	10,267	623	615	0.837	-	59		0.003
		Non-coronary revascularization	10,269	10,267	472	510	0.214	-	22		0.001
		Any revascularization	10,269	10,267	1058	1086	0.523	-	57		0.003
		Any major vascular event	10,269	10,267	2306	2312	0.920	-	111		0.005
I CARE [35]	Vitamin E *vs*. placebo	Primary composite	726	708	16	33	0.013	4	-	0.003	
		MI	726	708	7	17	0.039	1	-	0.001	
		Stroke	726	708	6	11	0.230	-	3		0.002
		CV death	726	708	3	5	0.501	-	3		0.002
PREDIMED^†^ [30]	MD + EVOO *vs*. usual diet	Rate of major CV events	2543	2450	96	109	0.254	-	11		0.002
	MD + nuts *vs*. usual diet	Rate of major CV events	2454	2450	83	109	0.056	-	1		0.0002
REDUCE-IT [36]	E-EPA *vs.* placebo	Composite of CV death, non-fatal MI/stroke, coronary revascularization, unstable angina	4089	4090	459	606	0.001	85	-	0.010	
R&P [32]	n-3 fatty acids *vs*. placebo	Primary composite endpoint	6239	6266	733	745	0.825	-	62		0.005
		Death from CV cause	6239	6,266	142	137	0.762	-	31		0.002
		Hospitalization for CV cause	6239	6266	620	630	0.835	-	58		0.005
TIDE [37]	Vitamin D *vs*. placebo	Primary CV outcome	607	614	2	3	1.000	-	7		0.006
		CV death	607	614	0	1	1.000	-	2		0.002
		Non-fatal MI	607	614	1	1	1.000	-	6		0.005
		Non-fatal stroke	607	614	1	1	1.000	-	6		0.005
		Any revascularization	607	614	5	7	0.773	-	5		0.004
		Hospitalization for heart failure	607	614	2	0	0.247	-	3		0.002
WAFACS [31]	ONS with folic acid, vitamins B_12_ and B_6_ *vs*. placebo	Combined major vascular disease	2721	2721	406	390	0.565	-	36		0.007
		MI	2721	2721	65	74	0.492	-	14		0.003
		Stroke	2721	2721	79	69	0.453	-	14		0.003
		Ischemic stroke	2721	2721	69	62	0.596	-	15		0.003
		Hemorrhagic stroke	2721	2721	10	6	0.454	-	4		0.001
		Coronary revascularization	2721	2721	253	255	0.963	-	40		0.007
		Coronary artery bypass grafting	2721	2721	87	98	0.455	-	16		0.003
		Percutaneous coronary intervention	2721	2721	192	177	0.450	-	22		0.004
		CV death	2721	2721	96	94	0.941	-	24		0.004
		MI, stroke and CV death	2721	2721	205	211	0.799	-	33		0.006
		Total CHD	2721	2721	283	280	0.929	-	41		0.008
		Total mortality	2721	2721	250	256	0.815	-	36		0.007

*ALA,* a-linolenic acid; *ASCEND*, A Study of Cardiovascular Events in Diabetes; *CHD,* coronary heart disease; *CV,* cardiovascular; *CVD,* cardiovascular disease; *DHA*, docosahexaenoic acid; *EPA*, eicosapentaenoic acid; *E-EPA*, Ethyl eicosapentaenoic acid; *EVOO*, extra-virgin olive oil; *FI*, fragility index; *FQ*, Fragility quotient; *MD,* Mediterranean diet; *MI*, Myocardial infarction; ONS, oral nutrient supplements; *PAD*, peripheral artery disease; *PREDIMED*, Prevención con Dieta Mediterránea; *RCT*, randomized controlled trial; *REDUCE-IT*, Reduction of Cardiovascular Events with Icosapent Ethyl–Intervention Trial*; RFI, reverse fragility index; RFQ*, reverse fragility quotient; *TIA*, transient ischemic attack; *TIDE*, the Thiazolidinedione Intervention with vitamin D Evaluation; *WAFACS,* the Women’s Antioxidant and Folic Acid Cardiovascular Study. ^†^ In the PREDIMED, the actual reported sample size in each arm was used for the FI and RFI calculations and not the person-years

The median calculated FI for the statistically significant comparisons was 3 (range; 1–85, IQR 1.85–2.4). In four (67%) out of six comparisons, the calculated FIs had values of 3. The median FQ was 0.004 (range: 0.001–0.010, IQR: 0.001–0.006).

The median RFI of the 53 non-statistically significant comparisons was 22 (range: 1–111, IQR: 6–40). In 16 (11%) out of 53 different comparisons, the RFI was ≤ 3. On the other hand, the median RFQ was 0.003 (range: 0.001–0.022, IQR: 0.002–0.006).

## Discussion

The present systematic review investigated RCTs which assessed the effects of nutritional interventions on CVOTs among patients with DM. The median FI of the comparisons was 3, indicating that the overall statistical significance hinges on 3 patient events solely. In 67% of the assessed comparisons, the FI had a value of ≤ 3, indicating that a different outcome for ≤ 3 patients could shift the statistical significance of the trial that is otherwise considered well-designed based on power calculations and sample size. On the other hand, the median RFI of the DM subgroups was equal to 8. Additionally, in 75% of the eligible trials, the number of patients lost to follow-up was greater than the calculated FI.

The median calculated FI herein (3) is comparable to the results of other studies across research fields and medical specialties such as critical care [41], ophthalmology [42], and urology [43]. It is, however, significantly lower when compared to the FI of DM treatment guidelines [44], which was calculated at 16, and to the median FI (26) of heart failure trials [45], thereby raising the already existing concerns in terms of reliability of the nutrition studies [19, 21, 46]. Interestingly, if available, the data of patients lost to follow-up might well reverse the statistical significance of the trial; hence, further questioning of the trials’ robustness arises. Notably, 20% of the RCTs did not report the number of patients lost to follow-up.

With regard to the RFI, in 71% of the trials, the RFI was lower than the number of patients lost to follow-up. This reveals an important point regarding the interpretation of statistical non-significance solely in the form of *P*-values since a different outcome for as few as five patients could reverse the results and lead to statistical significance. As previously mentioned, considerable advances in MNT make this an exciting time for the relatively young field of nutrition [47], elevating it to the rank of a fundamental component of quality DM care [4]. However, controversial results from epidemiologic studies during the last three decades have given rise to a critique of human nutrition research on numerous occasions [21, 48–53]. In fact, a large number of clinical nutrition RCTs have been published in recent years, many of which, unfortunately, created controversy [48–53].

Although nutrition remains the only etiological treatment for CVD, the present study revealed that only a limited amount of nutrition CVOTs with dichotomous primary outcomes were performed in patients with DM. This is probably due to the long duration required for a lifestyle change to alter hard dichotomous endpoints, the naturally slow disease progression, and the increasing attrition among patients required to comply with dietary modifications in long-duration studies [51]. Notably, most of the RCTs use intermediate biomarkers such as risk factors for CVD. Even fewer trials applied nutrition interventions solely, while a multidisciplinary approach, or a combination of diet and exercise, or hypoglycemic drugs, was more frequent. Moreover, as already mentioned, the majority of the RCTs involved interventions with supplements (fatty acids or vitamins), while only one study examined the effects of a dietary pattern (MD), this specifically being the PREDIMED diet. In addition, only a small proportion of the included trials demonstrated statistically significant results. In those trials, the low FI and the exceedingly large number of patients lost to follow-up indicate relatively low robustness, supporting the aforementioned increasing concerns regarding the reliability of the RCT design in nutrition research.

One issue with regard to the FI and the RFI is that they are both dependent on the number of events [21]. Nevertheless, a small number of events is common in CVOTs [54, 55] given their longitudinal design and increased expenses. Therefore, a relatively small number of events is also expected to occur in CVOTs with nutrition interventions.

Recently, the COMPAR-EU consortium has been developing a core outcome set for DM trials and self-managing and the results are awaited [56]. In parallel, during the year 2008, the Food and Drug Administration (FDA) issued the first guidelines on the design of CVOTs in DM, holding frequent Delphi-style panel discussions to confer on high-quality evidence concerning CVD, focusing mainly on newly developed glucose-lowering agents. Following the example of the FDA, the European Medicines Agency (EMA) issued similar requirements [57]. Although the majority of DM-specific outcomes involve linear variables (i.e., glucose levels), most of the CVOTs involve binary endpoints (i.e., stroke) [58]. In parallel, many of the trials included in the present analysis predated the CVOT requirements in that they included patients with DM without necessarily incorporating patients with relatively advanced disease, elderly patients, or people with some degree of renal impairment, as suggested for the CVOTs investigating medicines [59]. Moreover, the various CVOTs have revealed the vastness and complexity of CVD [54]. Comparisons between CVOTs should be performed with caution, considering the differences in sample characteristics, duration of DM, and severity of CV risk factors [59]. However, the total number of RCTs included in the present analysis was rather too small for the conduct of further meaningful comparisons. Although CVOTs in DM are designed to drive clinical practice changes, the low FIs and RFIs indicated herein do not commend nutrition interventions. Nevertheless, when following a more natural therapy such as a nutrition intervention, the side effects are often minimal, allowing for its prescription irrespectively of the magnitude of expected change.

The pivotal importance of multifactorial and comprehensive management of DM is highlighted in several clinical practice guidelines [60], indicating that lifestyle and medications should be paired for improved CV outcomes [61]. This is exemplified when considering that DM is often initiated by obesity; thus, any intervention targeting weight loss is more likely to improve CV outcomes compared to medication alone [61]. In the present analysis, none of the included RCTs involved an intervention with caloric restriction. This might partially explain the relatively low FI of the RCTs observed herein.

Our study has several limitations as the applicability of the FI itself has itself, by definition, important limitations. First, the FI can only be applied in clinical trials with binary outcomes and a 1:1 allocation ratio; therefore, clinically important continuous endpoints for CVD were excluded. Secondly, the FI does not account for the difference in outcome over time, therefore being inappropriate for time-to-event data and time-to-event statistical techniques used in some of the included trials. Furthermore, there is no specific cut-off value to classify robustness; thus, FI in isolation should be interpreted with caution and can provide very limited value. It is worth noting that since the FI and the RFI are absolute measures irrespective of a trial’s size, the use of the FQ and RFQ can aid in better understanding a trial’s robustness, considering the overall sample size [62, 63]. Lastly, only 50% of the included RCTs had a total sample size of patients with DM. Using the data for the DM subgroup for the analysis may have interfered with the randomization procedure and led to systematic bias, although a sensitivity analysis was performed to reduce this possibility. In parallel, the majority of the trials used a combined sample of patients with T1DM and T2DM without differentiating the results. If subgroup analyses for the two DM types had been feasible, the results might have been different.

Conversely, the findings of the FI analysis are consistent with other studies examining the FI of MD trials [21], peri-operative medicine [64], critical care [65], epilepsy [66], orthopedics [67], and other medical areas, reporting similarly low FIs. Moreover, the use of the RFI in a broader context can reduce the risk of overlooking advantageous interventions [18].

While acknowledging our study’s limitations, we advocate for the routine calculation of the FI in clinical trials as an aid in the interpretation of results. The design of a large, adequately powered clinical nutrition RCT researching hard endpoints is not always feasible. Therefore, dietary interventions tend to be used as complimentary rather than primary therapy. Moreover, clinical trials outcomes may be statistically significant by traditional statistical measures, but may lack clinical significance. Every research finding should be assessed by a physician before being implemented in clinical practice. The presentation of FI, in combination with the sample size and the number lost to follow-up, can facilitate a clinician’s appreciation not only of the robustness of statistically significant findings but also of the clinical meaningfulness, or lack thereof, of these findings.

## Conclusions

RCTs examining nutritional interventions and cardiovascular outcomes among patients with DM can be statistically fragile. Narrowing the scope of significance to metrics such as *p*-values and confidence intervals can lead to misinterpretations, selective reporting, and publication bias. FI and RFI can aid appraisal of statistically significant and non-significant results, respectively, as long as they are not interpreted as a measure of effect but as an additive perspective of a trial's weaknesses.

## Data Availability

All used data are presented in the manuscript.
